# Selenium Nanoparticles as a Natural Antioxidant and Metabolic Regulator in Aquaculture: A Review

**DOI:** 10.3390/antiox10091364

**Published:** 2021-08-27

**Authors:** Mahmoud A. O. Dawood, Mohammed F. El Basuini, Sevdan Yilmaz, Hany M. R. Abdel-Latif, Zulhisyam Abdul Kari, Mohammad Khairul Azhar Abdul Razab, Hamada A. Ahmed, Mahmoud Alagawany, Mahmoud S. Gewaily

**Affiliations:** 1 Animal Production Department, Faculty of Agriculture, Kafrelsheikh University, Kafr El-Sheikh 33516, Egypt; 2 The Center for Applied Research on the Environment and Sustainability, The American University in Cairo, Cairo 11835, Egypt; 3 Animal Production Department, Faculty of Agriculture, Tanta University, Tanta 31527, Egypt; m_fouad_islam@yahoo.com; 4 Faculty of Desert Agriculture, King Salman International University, South Sinai 46618, Egypt; 5 Department of Aquaculture, Faculty of Marine Sciences and Technology, Canakkale Onsekiz Mart University, Canakkale 17100, Turkey; sevdanyilmaz@comu.edu.tr; 6 Department of Poultry and Fish Diseases, Faculty of Veterinary Medicine, Alexandria University, Behera 22758, Egypt; hmhany@alexu.edu.eg; 7 Faculty of Agro-Based Industry, Jeli Campus, Universiti Malaysia Kelantan, Jeli 17600, Malaysia; 8 School of Health Sciences, Universiti Sains Malaysia, Health Campus, Kubang Kerian 16150, Kelantan, Malaysia; 9 Department of Nutrition and Veterinary Clinical Nutrition, Faculty of Veterinary Medicine, Damanhour University, Damanhour 22511, Egypt; hamada_nutrition@yahoo.com; 10 Poultry Department, Faculty of Agriculture, Zagazig University, Zagazig 44511, Egypt; dr.mahmoud.alagwany@gmail.com; 11 Department of Anatomy and Embryology, Faculty of Veterinary Medicine, Kafrelsheikh University, Kafr El-Sheikh 33516, Egypt; drmahmoud_gewaily@yahoo.com

**Keywords:** trace elements, nanotechnology, physiological function, metabolism, biological roles, aquaculture

## Abstract

Balanced aquafeed is the key factor for enhancing the productivity of aquatic animals. In this context, aquatic animals require optimal amounts of lipids, proteins, carbohydrates, vitamins, and minerals. The original plant and animals’ ingredients in the basal diets are insufficient to provide aquafeed with suitable amounts of minerals. Concurrently, elements should be incorporated in aquafeed in optimal doses, which differ based on the basal diets’ species, age, size, and composition. Selenium is one of the essential trace elements involved in various metabolic, biological, and physiological functions. Se acts as a precursor for antioxidative enzyme synthesis leading to high total antioxidative capacity. Further, Se can enhance the immune response and the tolerance of aquatic animals to infectious diseases. Several metabolic mechanisms, such as thyroid hormone production, cytokine formation, fecundity, and DNA synthesis, require sufficient Se addition. The recent progress in the nanotechnology industry is also applied in the production of Se nanoparticles. Indeed, Se nanoparticles are elaborated as more soluble and bioavailable than the organic and non-organic forms. In aquaculture, multiple investigations have elaborated the role of Se nanoparticles on the performances and wellbeing of aquatic animals. In this review, the outputs of recent studies associated with the role of Se nanoparticles on aquatic animals’ performances were simplified and presented for more research and development.

## 1. Introduction

Nanotechnology is a growing technology with high potential for application in the aquaculture industry [1]. Nanoengineered minerals (<100 nm) are well recognized in aquafeed due to their high solubility, active surface, and functionality [2]. Nano minerals are characterized by higher surface area affinity, higher solubility, thermal resistance, low toxicity, slow excretion rate, and sustained release [3]. Accordingly, nanominerals can beneficially affect animals’ metabolic, physiological, and biological functions [4]. Selenium (Se) particles are one of the microelements involved in various tasks in the entire body of aquatic animals [1,5]. The targeting role of Se is to protect cells from oxidation through the formation of antioxidative defenses [6]. Se also acts as a cofactor in the formation of selenoproteins involved in the catalysis of hydroperoxide [7]. Besides, Se contributes to the metabolism of the thyroid gland, reproduction, and development of body tissues [8]. Initially, Se has organic and non-organic forms with low bioavailability, solubility, and adherence properties [9,10]. Thus, introducing Se nanoparticles in the aquafeed industry is highly recommended to maximize aquatic animals’ health status and productivity.

Se nanoparticles are well investigated with high potential as a growth promotor, antioxidative, and immunostimulant agent in aquaculture [11,12]. Multiple studies reported the necessity of including Se nanoparticles for enhancing the growth performance, physiological, and health status in aquatic animals [5,13,14]. Due to the low margin between the benefits and the toxicity, Se nanoparticles have to be included in aquafeed based on a dose-specific manner. Excessive levels of dietary Se (>20–30 mg/kg) are hazardous to most animals, including livestock and fish [15]. The novel Se nanoparticles are characterized by their low toxicity and high functionality [1]. Markedly, the inclusion of Se nanoparticles led to enhancement in aquatic animals’ growth, performance, and productivity [10,16,17]. In this article, the multiple roles of Se nanoparticles are presented and discussed with particular focus on (1) the growth-promoting and feed utilization effects, (2) the metabolic regulation roles, (3) the antioxidative and physiological aspects, and (4) the Se nanoparticles role as antistress agent.

## 2. Selenium Source, Forms, and Availability

Microelements are required at adequate levels and forms to contribute to several metabolic, physiological, and biological functions in the entire body of the organisms [18,19]. Zinc, copper, and selenium are among these microelements which should be fortified in fish diets to guarantee optimum growth and wellbeing [20]. A particular focus has been given to selenium (Se) due to its crucial role in antioxidative capacity, male reproduction, anti-carcinogenesis, thyroid metabolism, and muscle development [4]. Se is firstly introduced to the scientific community in 1818 by Jacob Berzelius and has two primary forms (organic and non-organic) [1]. The inorganic Se includes selenite (Se^4+^), selenide (Se^2−^), and selenate (Se^6+^), while the organic form of Se such as yeast-Se [21]. Se functionality depends on its form, and organic Se is more bioavailability than inorganic Se sources [9,22,23]. However, organic and inorganic Se sources are less water soluble, permeable, and bioavailable for metabolic and physiological functions [1]. The low surface adherence aptitudes lower its activity in the animal’s body. Regarding the advanced steps that have been investigated in applying nanotechnology in several fields, including medication, food, and feed preparation [24], Se nanoforms are suggested as an active source for aquafeed [12]. Se nanoforms are more biologically available with fewer amounts making them less toxic and highly effective [25]. Further, Se nanoforms have a wide surface area that is possibly responsible for their functionality and permeability [11].

## 3. The Preparation of Selenium Nanoforms

Nanoengineered minerals are approved in the modern-day sciences and can be produced by several techniques. Indeed, nanosynthesized Se is known for its bio-physiological properties involved in the antioxidation and antibacterial potential [24]. Nanoparticles of Se can be produced by chemical, physical, and biological techniques.

### 3.1. Physical Form

The physical technique depends on microwave irradiation, laser ablation, and hydrothermal methods [26]. The atomic force microscopy is used to quantify the laser-ablated samples placed on silicon wafers, glass, and metallic gold sheets. The laser ablation process entails separating Se from a solid surface using a high-intensity laser pulse energy, transforming Se into plasma [27]. Upon mutual contact, plasma carrying a high concentration of Se ions aggregates into minute embryonic nuclei, thereby developing and stabilizing slowly. Nano Se can be produced through the hydrothermal route, using a nucleation-dissolution-recrystallization-based growth mechanism [28]. Briefly, sodium hydroxide (2.4 M), sodium selenite (0.5 mM), and sodium formate (2 mM) can be mixed and added to the autoclave at 100 °C for 25 h. Then, a considerable number of dark-gray color-floated Se nanoparticles would appear and can be easily collected.

### 3.2. Chemical Form

In the chemical technique, nano Se particles require different reductants using Se precursor salts. In this method, sodium selenite and ascorbic acid are used to produce Se nanoparticles which can be observed in a light orange color [29]. Ascorbic acid is also known for its functionality as antioxidative and immunostimulation roles. Furthermore, nano Se form (35–70 nm) was formed chemically in ambient circumstances with organic acids such as acetic and oxalic acid and polyvinyl alcohol (PVA) as a binding material [30]. A carboxylic group of organic acids was identified to decrease Se salt to nanoparticles during its acid-induced production. Other carboxyl groups, including benzoic acid and gallic acid, can be employed instead of acetic acid [31]. In another method, Se nanoparticles were produced by mixing sodium selenite and glucose in water followed by 20 min vigorous stirring [32,33]. The aldehyde group of glucose is supposed to be oxidized to the carboxyl group by the nucleophilic addition of hydroxyl group (OH), resulting in Se nanoparticles.

### 3.3. Biological Form

The biological method used to produce Se nanoform is widely validated due to its easy processing, low toxicity impacts, and high pharmacological merits [34]. In this method, yeast, bacteria, and fungi were used with plant origin materials [35]. Using *Vitis vinifera* (raisin) fruit and selenous acid, Se nanoparticles were made by immersing shed dry fruits overnight and crushing them, then removing impurities in distilled water for 30 min [36]. The filtered solution was mixed with a selenous acid solution and centrifuged for 15 min to produce nanoparticles with a spherical shape. Raisins include carbohydrates, flavonoids, vitamins, and other compounds that may function in nanoparticle formation (3–18 nm) [36]. In another trial, Se nanoparticles (20–50 nm) were produced using Spirulina polysaccharides with the “Solution phase method” [37]. Sodium selenite was mixed Spirulina polysaccharide solution then the ascorbic acid solution was added. The produced Se nanoparticles were actively involved in anti-cancer activity when tested against A375 human myeloma cell lines. *Klebsiella pneumoniae* bacteria were used to produce the Se nanoform using Se chloride in tryptic soy broth (TSB) culture media [38]. *Shewanella* sp. HN41 was also used for Se nanoparticle production using lactate and selenite [39]. Besides, *Bacillus* sp. JAPSK2 [40] and *Zooglea ramigera* [41] are used to produce Se nanoparticles using Se chloride and Se oxyanions, respectively. Sodium selenite was also used to produce Se nanoparticles by *Pantoea agglomerans* bacteria [42]. Faramarzi et al. [43] reported that Se nanoparticles could be produced by yeast (*Saccharomyces cerevisiae*). Several other bacterial strains, including *Streptococcus thermophilus*, *Lactobacillus casei*, and *Lactobacillus acidophilus*, are successfully used to produce Se nanoparticles [44]. Generally, the bioactivity of bacterial strains in Se nanoform production depends mainly on the pH and growing media.

## 4. The Role of Selenium Nanoparticles on the Growth Performance

The growth performance of aquatic animals depends on several factors such as well management, water quality, vaccination, and temperature [45] (Table 1). Besides, nutritionally balanced aquafeed is another vital factor associated with improving the feed digestibility and thereby the health condition and growth performance of finfish species [46]. Optimum feed formulations should contain both macro and microelements to fulfill the basic requirements of finfish species. Over or low levels of these elements cause impaired metabolic and physiological functions and led to malnutritional features. Nutritionally, Se can stimulate growth hormone production, leading to high growth performance in fish [12]. Se bind with deiodinase enzyme, which is required for thyroid hormone regulation [47]. Fish, in the same way as other vertebrates, have a pituitary gland involved in the secretion of thyroid hormones that stimulate the secretion of growth hormones [8]. In this regard, Khan et al. [48] elucidated that *Tor putitora* fed dietary Se nanoparticles showed increased growth hormone levels. Asian seabass (*Lates calcarifer*) fed dietary nano Se at 4 mg/kg showed enhanced growth performance in a feeding trial that lasted for six weeks [49]. In another trial that lasted for four weeks, Asian seabass-fed nano Se at 4 mg/kg showed enhanced growth and survival rates. The inclusion of dietary Se nanoparticles at 3 mg/kg in the diets of early weaning gilthead seabream (*Sparus aurata*) improved larval and growth performance [50]. The authors stated that the role of Se nanoparticles in improving larval growth is related to bone mineralization and the prevention of skeleton anomalies. Dawood et al. [51] also reported that red sea bream (*Pagrus major*) fed dietary nano Se at 1 mg/kg had enhanced growth performance and feed utilization. The authors correlated the enhanced growth performance with the role of Se nanoparticles in activating the protease, thereby feed utilization. In several feeding trials that lasted for 4 to 8 weeks, Nile tilapia (*Oreochromis niloticus*) fed dietary nano Se at 1–2 mg/kg showed enhanced growth performance and feed utilization [25,52,53,54,55]. Notably, Abd El-Kader et al. [56] and Abd El-Kader et al. [57] reported that European seabass (*Dicentrarchus labrax*) fed Se nanoparticles at 0.5–1 mg/kg had enhanced growth performance and feed efficiency. The authors attributed enhance growth performance of European seabass to the role of nano Se in the upregulation of Insulin-like growth factor 1 (*IGF-1*) gene expression. In a feeding trial that lasted for 70 days, Khan et al. [48] and Khan et al. [58] reported that Mahseer fish displayed increased growth performance. Kumar et al. [59] and Kumar et al. [60] stated that *Pangasinodon hypophthalmus* treated with Se nanoparticles at 1–2 mg/kg had enhanced growth performance. Further, rainbow trout (*Oncorhynchus mykiss*) fed dietary Se nanoparticles at 2 mg/kg for 60 days had enhanced growth performance and feed utilization [61]. In a nine-week feeding trial, Goldfish (*Carassius auratus*) fed dietary Se nanoparticles at 0.6 mg/kg and enhanced weight gain, specific growth rates, and *IGF-1* gene expressions [62]. Jahanbakhshi et al. [62] elucidated the enhanced growth performance of Goldfish to the role of Se nanoparticles in improving ghrelin hormone and improving feed utilization. In common carp (*Cyprinus carpio*), Saffari et al. [63] and Ashour et al. [64] reported that the inclusion of dietary nano Se at 0.7–1 mg/kg resulted in improved growth performance and feed utilization. Rohu (*Labeo rohita* Hamilton) fed dietary Se nanoparticles at 0.3 mg/kg for 120 days had enhanced growth performance [65]. Liu et al. [66] reported that Grass carp (*Ctenopharyngodon idella*) fed dietary Se nanoparticles at 0.6–0.9 mg/kg for ten weeks had enhanced growth performance and survival rate. On the other hand, rainbow trout (*O. mykiss*) fed dietary Se nanoparticles at 1 mg/kg had no marked effects on the growth performance [67].

Based on the studies mentioned above, Se nanoparticles can enhance the growth performance of aquatic animals. The role of Se nanoparticles is probably attributed to the effect of Se in activating the digestive enzymes and enhancing the integrity of intestinal villi [45]. It should be noted that Ghazi et al. [55] reported enhanced intestinal morphometry, villi length, and goblet cells number in Nile tilapia fed Se nanoparticles. This study confirms that Se nanoparticles have a notable role in improving intestinal health, leading to high feed utilization and thereby improved growth performance. Further, the Se nanoform has active antibacterial potential that may inhibit the growth of pathogenic microorganisms in the intestines of aquatic animals. Accordingly, the beneficial bacteria can perform more effectively in digesting the nutrients through the secretion of digestive enzymes. The enhancement in the growth performance of fish is also explained by Ibrahim et al. [5], who indicated that Nile tilapia-fed dietary Se nanoparticles (0.4–0.8 mg/kg) showed enhanced growth performance and feed efficiency. The authors also confirmed that Se nanoparticles caused a positive impact on the intestinal histomorphological features. Increased villi length and width, a high number of goblet cells, and marked villi branching and integrity were also seen in Nile tilapia fed Se nanoparticles. Indeed, Se acts as a precursor for synthesizing selenoproteins involved in high protein levels in the intestinal villi, leading to increased digestive enzyme activity, thereby high feed utilization, metabolic function, and growth performance. Markedly, the nano form of Se is more efficient in enhancing the feed utilization in the entire body of fish associated with Se nanoparticles’ active surface and its small sizes that allow the particles to function with low amounts. Obviously, the inclusion of Se nanoparticles is recommended at 0.15–4 mg/kg depending on the fish species, feeding duration, and experimental conditions (Table 1).

## 5. Selenium Nanoparticles and the Antioxidative Capacity

Aquaculture activity is threatened with various stressors involved in reducing the health status of aquatic animals [73]. Aquatic animals suffer from biotic and abiotic stressors which impair their biological and physiological functions. Stressors disrupt the antioxidative balance due to the high generation of reactive oxygen metabolites (ROS), hydrogen peroxide, and peroxide radicals [74]. These free radicals induce lipid peroxidation and led to DNA and cell damage. Severe oxidative stress is the precursor for several antioxidative responses such as superoxide dismutase (SOD), catalase (CAT), glutathione-S-transferase (GST), and peroxidase (GPx) [75]. Se is known for its role in forming selenoproteins that help synthesize glutathione peroxidase enzymes [7]. More specifically, Se nanoparticles are noted to upregulate the expression of GPx by forming selenophosphate [8]. Therefore, Se nanoparticles are suggested as a powerful antioxidative agent in aquaculture.

Longbaf Dezfouli et al. [49] reported that Asian seabass-fed dietary Se nanoparticles (4 mg/kg) showed improved antioxidative capacity (SOD, CAT, and GPx). Further, Dawood et al. [51] reported that red sea bream-fed dietary Se nanoparticles (1–2 mg/kg) had enhanced CAT. Besides, Dawood et al. [25] stated that Nile tilapia-fed dietary Se nanoparticles (1 mg/kg) had enhanced GPx, SOD, and CAT. Additionally, Al-Deriny et al. [52] reported that Nile tilapia-fed Se nanoparticles (1 mg/kg) had activated SOD. In another study by Ghazi et al. [55], Nile tilapia treated with Se nanoparticles (1 mg/kg) had enhanced SOD and CAT and reduced malondialdehyde levels (MDA). In European Seabass, Abd El-Kader et al. [56] and Abd El-Kader et al. [57] illustrated high SOD and CAT and low MDA levels in fish treated with Se nanoparticles (0.5–1 mg/kg). Mahseer fish fed dietary Se nanoparticles (0.68 mg/kg) had enhanced GPx activity. Markedly, *P. hypophthalmus* fed dietary Se nanoparticles (1–2 mg/kg) for 95 days had enhanced CAT, GST, and GPx [70]. Kohshahi et al. [67] indicated that rainbow trout-fed Se nanoparticles (1 mg/kg) had enhanced GPx activity. Seyedi et al. [72] reported that Goldfish fed Se nanoparticles (1 mg/kg) for 60 days had enhanced GPx. Saffari et al. [63] and Ashouri et al. [64] reported that common carp-fed dietary Se nanoparticles (0.7–1 mg/kg) had enhanced SOD, CAT, and GPx and reduced MDA levels. Furthermore, rohu fish fed Se nanoparticles (0.3 mg/kg) [65] and Crucian carp [22] fed 0.5 mg Se nanoparticles/kg showed enhanced SOD and GPx, respectively.

It is evident that Se nanoparticles can enhance the antioxidative capacity of aquatic animals through the activation of SOD, CAT, GST, and GPx enzymes. In addition, Se nanoparticles reduced lipid peroxidation through the reduction of MDA levels.

## 6. Effect of Selenium Nanoparticles on the Immunological, Biochemical, and Hematological Parameters of Blood

Aquatic animals suffer from various biotic and abiotic stressors that may occur during the farming season [76]. Nutritionally balanced aquafeed is the main key factor that helps counteract these stressors leading to high productivity and well-being [5]. Microelements, including Se, is another vital strategy to guarantee the balance of the nutritional value of aquafeed [1]. Se has several physiological roles in animals’ bodies as an antioxidant and a metabolic and immunostimulant agent [69]. Indeed, Se is a precursor for several metabolites involved in several physiological functions in the entire body [8]. Se is involved in the synthesis of selenoproteins which contribute to antioxidative and immune systems [47]. Besides regulating hepatic and renal functions as vital tissues in the detoxification and releasing body toxicants and nitrogen residuals [74]. Furthermore, hematological and blood biochemical indices are markedly influenced by the nutritional value of aquafeed and its content of Se.

In this regard, Asian seabass-fed dietary Se nanoparticles (4 mg/kg) showed reduced alanine aminotransferase (ALT) and aspartate transaminase levels (AST) [49]. Further, Deilamy Pour et al. [14] stated that Asian seabass-fed Se nanoparticles reduced glucose, cholesterol, triglyceride, protein indices, immunoglobulin, IgM, C3, and ACH50 indexes. In red sea bream, Dawood et al. [51] reported that Se nanoparticles (1 mg/kg) resulted in increased hematocrit and biological antioxidant potential and reduced reactive oxygen metabolites, cholesterol, and triglycerides. In another study, Dawood et al. [68] concluded that Se nanoparticles resulted in the increased alternative complement pathway, nitro blue tetrazolium activity (NBT), total serum protein, bactericidal activity, lysozyme activity, and skin mucus secretions in red sea bream. In Nile tilapia, Ayoub et al. [53] reported enhanced serum lysozyme and respiratory burst activities by dietary Se nanoparticles (1–2 mg/kg). Further, Abu-Elala et al. [54] and Ghazi et al. [55] reported that Nile tilapia-fed dietary Se nanoparticles (1–2 mg/mg) showed enhanced phagocytic, lysozyme activities, phagocytic index, red blood cells, globulin, and immunoglobulin M. In European seabass, Abd El-Kader et al. [56] and Abd El-Kader et al. [57] reported increased hemoglobin, hematocrit, red blood cells, white blood cells, total serum protein globulin, phagocytic index, phagocytic, and lysozyme activities by dietary Se nanoparticles (0.5–1 mg/kg). Concurrently, Khan et al. [48] and Khan et al. [58] reported that Mahseer fish fed dietary Se nanoparticles (0.68 mg/kg) had increased red blood cell count, hemoglobin level, hematocrit values, and lysozyme activity. Kumar et al. [60] reported that *P. hypophthalmus* fed dietary Se nanoparticles (0.5 mg/kg) had increased acetylcholine esterase, NBT, total immunoglobulin, myeloperoxidase, globulin, and reduced serum cortisol, lipid peroxidation, and blood glucose. Nazer et al. [61] reported that rainbow trout fed dietary Se nanoparticles (2 mg/kg) had increased red blood cells, hemoglobin, and hematocrit. Saffari et al. [63] stated that Se nanoparticles increased AST, alanine transaminase, and lactate dehydrogenase activity in common carp. Further, Ashouri et al. [64] elucidated that common carp fed Se nanoparticles had increased total protein, globulin, serum high-density lipoprotein, and reduced albumin, AST, and ALT. In Rohu, Swain, et al. [65] reported that Se nanoparticles (0.3 mg/kg) resulted in an increased respiratory burst, lysozyme, acetylcholine esterase activity, myeloperoxidase activities, and reduced lactate dehydrogenase and alkaline phosphatase activities. In grass carp, Liu et al. [66] stated the role of Se nanoparticles in improving the liver fatty acid oxidation and triglyceride hydrolyses genes (*PPARα*, *CPT1*, *ATGL*, and *LPL*) and high-density lipoprotein cholesterol and reduced serum triglyceride and total cholesterol, ALT, and AST. The role of Se nanoparticles contributed to the regulation of several physiological functions in aquatic animals. The functions are related to the liver, kidney, spleen, and immune responses related to Se nanoparticles in aquafeed.

## 7. Selenium Nanoparticles against Stressful Conditions in Aquaculture

Stressful conditions, including suboptimal and over optimal water temperature, high ammonia levels, high stocking density, and infection with pathogenic invaders, commonly exist in aquaculture [77]. The continuous exposure to these stressors led to impaired health status and low production. Indeed, stressful conditions are involved in the imbalance of the physiological and metabolic function of a fish’s entire body [78]. Along with well management practices, high-quality feeds containing macro and micronutrients are recommended to enable fish to perform well and resist biotic and abiotic stressors [5]. In this context, red sea bream fed Se nanoparticles (1–2 mg/kg) showed high resistance against low salinity stress [68]. *P. hypophthalmus* treated with 1–2 mg/kg of Se nanoparticles showed high resistance to thermal stress [59]. Further, rainbow trout fed Se nanoparticles (2 mg/kg) showed had high resistance to sublethal ammonia stress [61]. Dietary Se nanoparticles (1 mg/kg) relieved the impacts of malathion-induced stress in Caspian roach [71]. Abu-Elala et al. [54] illuminated that dietary Se nanoparticles (1 mg/kg) accelerated cadmium toxicity-induced inflammation and immunosuppression in Nile tilapia. Furthermore, Ayoub et al. [53] showed that Nile tilapia-fed dietary Se nanoparticles (1–2 mg/kg) had high resistance against *Aeromonas sobria* infection. Rathore et al. [12] reported that Nile tilapia treated with Se nanoparticles have high resistance against *A. hydrophila* infection. *P. hypophthalmus* fed dietary Se nanoparticles (1–2 mg/kg) also showed high resistance against *A. veronii* biovar *sobria* [17]. In addition, rohu treated with 0.3 mg Se nanoparticles/kg had increased resistance against *A. hydrophila* infection [65]. The protective role of Se in relieving stress-induced inflammation, oxidative stress, and immunosuppression is probably attributed to the antioxidative and the regulation of stress-related genes. In this regard, Se nanoparticles are illustrated to reduce the expression of heat shock protein 70 (*HSP70*) as a stress-related marker in Nile tilapia [52], European Seabass [56,57], and *P. hypophthalmus* [60].

## 8. Toxicity of Selenium Nanoparticles

As mentioned earlier, selenium nanoparticles are required in optimum doses to formulate nutritionally balanced aquafeed [4,79]. Nevertheless, over-dosing and high levels of Se nanoparticles could induce toxicity and impairment of several physiological and biological functions in fish bodies [80]. Geological, industrial, and agricultural activities are the primary resources for over Se levels in the water and ecosystems [81]. Besides the rising and frequent use of these nanoparticles, concern over their toxicity in aquatic ecosystems and at cross-trophic levels in the food chain is growing [82]. Accordingly, the release of high amounts of Se in the ecosystem could induce toxicity and oxidative stress in aquatic animals. Bioaccumulation of Se nanoparticles can reach the fish through the gills and orally through the intestines [1]. The synthesis of methyl-selenide, a part of superoxide radicals’ formation, is the significant toxic impact of Se bioaccumulation [83]. In addition to producing free radicals, Se has an inhibiting effect on thiol proteins, which have an antioxidant function. Furthermore, high levels of accumulated Se nanoparticles in the fish tissues and fluids (muscles, intestines, livers, kidneys, and blood) can indirectly reach the animals and human body, causing severe toxicity [84]. The impact of Se nanoparticles on the productivity and health status of aquatic animals is scarcely investigated. In this regard, a high dose of Se nanoparticles caused deleterious effects on the gills and liver histopathology as well as the related metabolic indices (e.g., liver function (ALP, AST, and ALT), LDH, and acetylcholine esterase (AChE)) in *P. hypophthalmus* [85]. Toxicity with Se nanoparticles in goldfish (*Carassius auratus*) increased MDA levels and GPx in seminal plasma and DNA damage of sperm and increased spermatocyte and spermatid [72].

## 9. Conclusions and Future Perspectives

Nanotechnology, an innovative technology, is gaining attraction worldwide because of its potential for medication delivery and nutritional enhancement. Se nanoparticles have high potential as antibacterial, antioxidants, and growth-promoting effects. In the aquaculture industry, optimal feeds are the most significant factor for optimizing the nutritional requirements of aquatic animals. Indeed, nutritionally balanced aquafeed should contain suitable amounts of Se with bioavailable properties. Se nanoparticles can easily be absorbed and cross the intestinal barriers of the local intestines of aquatic animals. Concurrently, Se would be available for several biological, metabolic, and physiological functions involved in metabolism, antioxidation, and immunity. Furthermore, Se nanoparticles are unique molecules targeting the pathogenic microorganisms that lead to high local intestinal immunity and digestion capacity, thereby increasing the performance and productivity of aquatic animals. Although the present article presents a plethora of outputs of relevant investigations in aquatic animals, future studies are required to understand Se nanoparticles’ specific mode of action on the performances of aquatic animals.

## Figures and Tables

**Table 1:** The effects of selenium nanoparticles on the performances of aquatic animals.

Species	Dose	Duration	Effects	References
Asian seabass (*Lates calcarifer*)	4 mg/kg	6 weeks	Growth performance and immune response (↑) Alanine aminotransferase (ALT) and aspartate transaminase levels (AST) (↓) Liver superoxide dismutase (SOD), glutathione peroxidase (GPx), and catalase (CAT) (↔)	Longbaf Dezfouli, et al. [49]
Asian seabass (*Lates calcarifer*)	4 mg/kg	4 weeks	Growth performance, digestive enzymes, and lysozyme activity (↑) Serum levels of ALT, AST, ALP, and LDH (↔) Serum levels of glucose, cholesterol, triglyceride, protein indices, immunoglobulin, IgM, C3, and ACH50 indexes (↓)	Deilamy Pour et al. [14]
Gilthead seabream (*Sparus aurata*; Linnaeus, 1758)	3 mg/kg	24 days	Larval growth and bone mineralization and prevention of skeleton anomalies (↑)	Izquierdo, et al. [50]
Red seabream (*Pagrus major*)	1 mg/kg	45 days	The growth performance, feed efficiency, protease activity, hematocrit, and biological antioxidant potential (↑) Reactive oxygen metabolites, cholesterol, and triglycerides (↓)	Dawood, et al. [51]
Red seabream (*Pagrus major*)	1–2 mg/kg	45 days	Alternative complement pathway, nitro blue tetrazolium activity (NBT), total serum protein, CAT, serum bactericidal activity, serum lysozyme activity, and amounts of skin mucus secretions as well as stress resistance against low salinity stress (↑)	Dawood, et al. [68]
Nile tilapia (*Oreochromis niloticus*)	1 mg/kg	8 weeks	Growth performance, feed utilization, GPx, SOD, CAT, NBT, lysozyme, and phagocytosis activities, liver, and spleen *TNF-α* and *IL-1β* expressions upregulated (↑) Malondialdehyde (MDA) (↓)	Dawood, et al. [25]
Nile tilapia (*Oreochromis niloticus*)	1 mg/kg	60 days	Growth performance, feed efficiency, immunoglobulin M, SOD, and tumor necrosis factor-alpha (*TNF-α*) (↑) Heat shock protein 70 (*HSP70*) (↓)	Al-Deriny, et al. [52]
Nile tilapia (*Oreochromis niloticus*)	1–2 mg/kg	4 weeks	Serum lysozyme, respiratory burst activities, antioxidant enzymes, and resistance against *Aeromonas sobria* (↑)	Ayoub, et al. [53]
Nile tilapia (*Oreochromis niloticus*)	1 mg/kg	4 weeks	Growth indices, phagocytic, lysozyme activities, phagocytic index, *IGF-1*, *TNF-α*, *IL-1β*, *CAT* genes, resistance against cadmium toxicity (↑)	Abu-Elala, et al. [54]
Nile tilapia (*Oreochromis niloticus*)	1 mg/kg	60 days	Growth performance, intestinal morphometry, villi length, and goblet cells number hemoglobin, red blood cells, globulin, phagocytic activity, phagocytic index, lysozyme activity, immunoglobulin M, SOD, and CAT (↑) FCR and MDA (↓)	Ghazi, et al. [55]
European Seabass (*Dicentrarchus labrax*)	0.5–1 mg/kg	90 days	Growth performance, feed efficiency, Hb, PCV, RBCs, WBCs, total serum protein globulin, phagocytic index, phagocytic, lysozyme activities, SOD, CAT, *GH*, *IGF-1*, *IL-8*, and *IL-1β* (↑) MDA and *HSP70* (↓)	Abd El-Kader et al. [57] and Abd El-Kader et al. [56]
Mahseer fish (*Tor putitora*)	0.68 mg/kg	70 days	Growth performance, red blood cell count, hemoglobin level, hematocrit values, lysozyme activity, serum growth hormone levels, tissue total protein content, and GPx (↑)	Khan et al. [48] and Khan et al. [58]
*Pangasinodon* *hypophthalmus*	1–2 mg/kg	60 days	Growth performance, antioxidative status, immunity, and neurotransmitter enzyme activity (↑)	Kumar et al. [59]
*Pangasinodon* *hypophthalmus*	1–2 mg/kg	72 days	Thermal tolerance (↑)	Kumar et al. [69]
*Pangasinodon* *hypophthalmus*	1–2 mg/kg	60 days	Resistance against *Aeromonas veronii* biovar *sobria* (↑)	Kumar and Singh [17]
*Pangasinodon* *hypophthalmus*	1–2 mg/kg	95 days	CAT, glutathione-s-transferase (GST), and GPx (↑)	Kumar et al. [70]
*Pangasianodon* *hypophthalmus*	0.5 mg/kg	2 months	Growth performance, anti-oxidative status, acetylcholine esterase, NBT, total immunoglobulin, myeloperoxidase, globulin, and high-temperature resistance (↑) Serum cortisol, lipid peroxidation in the liver, gill, and kidney, blood glucose, *HSP70* in gill and liver (↓)	Kumar, et al. [60]
Rainbow trout (*Oncorhynchus mykiss*)	1 mg/kg	8 weeks	GPx (↑) The survival rate, growth performance, feed utilization, and body composition (↔)	Kohshahi, et al. [67]
Rainbow trout (*Oncorhynchus mykiss*)	2 mg/kg	-	Percentage of egg hatching (↑)	Mahdave Jehanabad, et al. [16]
Rainbow trout (*Oncorhynchus mykiss*)	2 mg/kg	60 days	Growth parameters, RBC, hemoglobin, hematocrit, and resistance against sublethal ammonia stress (↑) WBC (↔)	Nazer, et al. [61]
Caspian roach (*Rutilus caspicus*)	1 mg/kg	28 days	Resistance to malathion stress (↑)	Zahmatkesh, et al. [71]
Goldfish (*Carassius auratus*)	0.6 mg/kg	9 weeks	Weight gain, specific growth rates (SGR), mucosal immunity, ghrelin, and *IGF-1* genes expressions (↑) FCR (↓)	Jahanbakhshi et al. [62]
Goldfish (*Carassius auratus*)	1 mg/kg	60 days	Male semen quality and GPx (↑)	Seyedi, et al. [72]
Common carp (*Cyprinus carpio*)	0.7 mg/kg	8 weeks	Growth performance, liver GPx, SOD, and CAT activities (↑) MDA, AST, alanine transaminase, and lactate dehydrogenase activity (↓)	Saffari, et al. [63]
Common carp (*Cyprinus carpio*)	1 mg/kg	8 weeks	Growth performance, GPx, SOD, total protein, globulin, serum high-density lipoprotein (HDL) (↑) Albumin, MDA, AST, and alanine transaminase (ALT) activities (↓) Feed efficiency (↔)	Ashouri et al. [64]
Rohu (*Labeo rohita* Hamilton)	0.3 mg/kg	120 days	Growth performance, respiratory burst, lysozyme, SOD, acetylcholine esterase activity, myeloperoxidase activities, resistance *Aeromonas hydrophila* (↑) Lactate dehydrogenase and alkaline phosphatase activities (↓)	Swain, et al. [65]
Crucian carp (*Carassius auratus* gibelio)	0.5 mg/kg	30 days	GPx in plasma and liver (↑)	Zhou et al. [22]
Grass carp (*Ctenopharyngodon idella*)	0.6–0.9 mg/kg	10 weeks	Growth performance, survival rate, liver fatty acid oxidation, and triglyceride hydrolyses genes (*PPARα*, *CPT1*, *ATGL*, and *LPL*) and high-density lipoprotein cholesterol (↑) Serum triglyceride and total cholesterol, ALT, and AST (↓)	Liu et al. [66]
Whiteleg shrimp (*Litopenaeus vannamei*)	0.15 mg/kg	56 days	Growth performance (↑) FCR (↓)	Karamzadeh et al. [13]

Variation in the treated fish compared to controls: (↑), significantly increases; (↓), significantly decreased; (↔), no significant change.
